# Supplementary material for: A Global Screen for Assembly State Changes of the Mitotic Proteome by SEC-SWATH-MS
Source: Cell Syst. 2020 Feb 26;10(2):133–155.e6. doi: 10.1016/j.cels.2020.01.001 (PMC7042714; doi:10.1016/j.cels.2020.01.001)

Q7LGA3 | HS2ST_HUMAN | HS2ST1 HS2ST KIAA0448
Monomer MW [kDa]: 41.881 Monomer expected elution fraction: 48

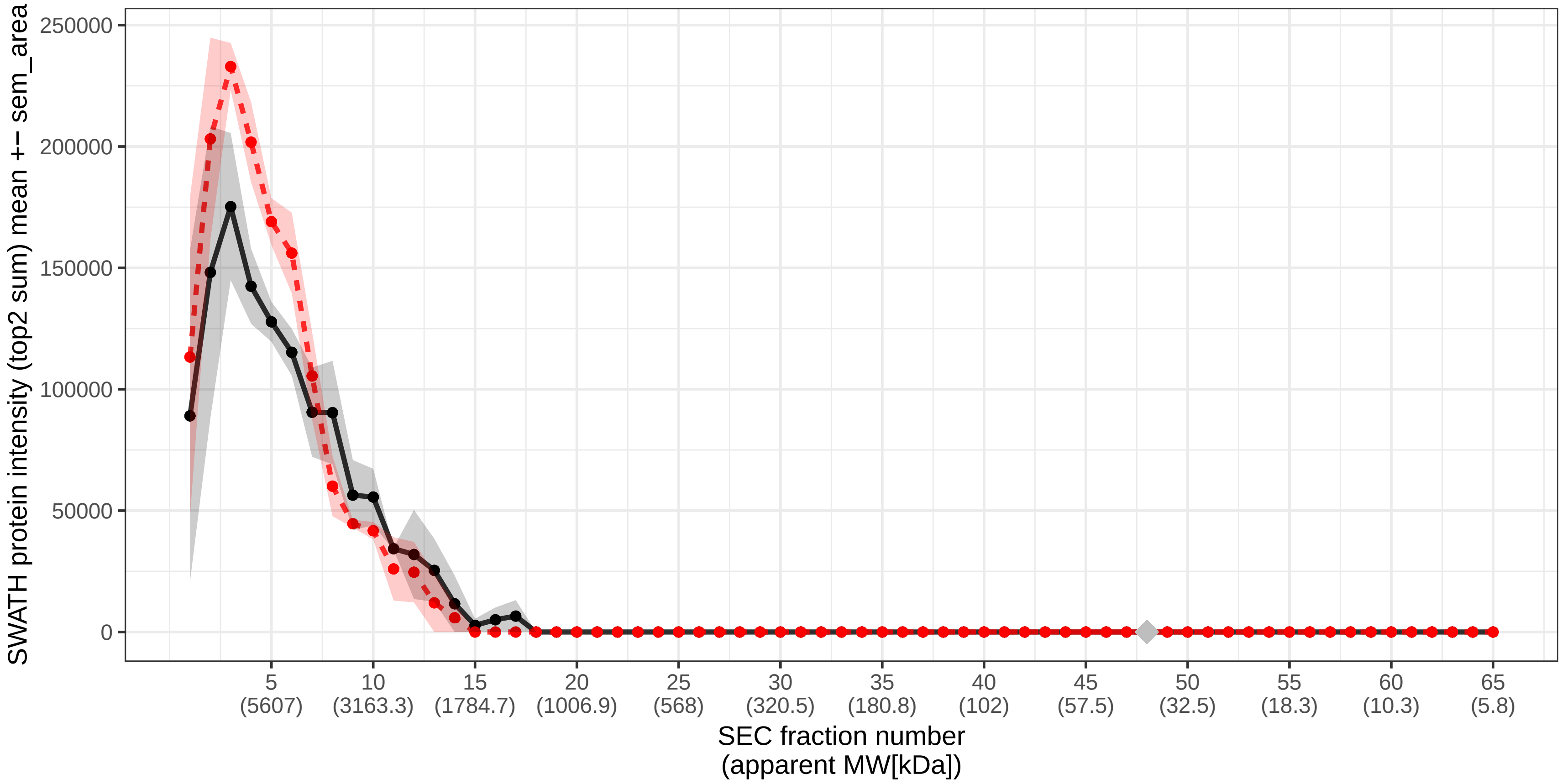

Supplement: Data S1. SEC-SWATH-MS Protein Chromatograms, Related to Figure 1 [file mmc6.zip › SECchrom_Q7LGA3_HS2ST_HUMAN_HS2ST1_HS2ST_KIAA0448.pdf]
